# Open-Label, Randomized, Two-Way, Crossover Study Assessing the Bioequivalence of the Liquid Formulation versus the Freeze-Dried Formulation of Recombinant Human FSH and Recombinant Human LH in a Fixed 2:1 Combination (Pergoveris^®^) in Pituitary-Suppressed Healthy Women

**DOI:** 10.3389/fendo.2017.00371

**Published:** 2018-01-11

**Authors:** Wilhelmina Bagchus, Özkan Yalkinoglu, Peter Wolna

**Affiliations:** ^1^Merck Institute for Pharmacometrics, Lausanne, Switzerland, an Affiliate of Merck KGaA, Darmstadt, Germany; ^2^Merck KGaA, Darmstadt, Germany

**Keywords:** recombinant human follicle-stimulating hormone, recombinant human luteinizing hormone, pharmacokinetics, bioequivalence, liquid formulation, freeze-dried formulation, safety

## Abstract

This was a Phase I, open-label, randomized, two-period, two-sequence crossover study [ClinicalTrials.gov NCT02317809 (https://www.clinicaltrials.gov/ct2/show/NCT02317809); EudraCT 2014-003506-32] assessing the bioequivalence of the liquid and freeze-dried formulations of fixed-dose, fixed-ratio (2:1) combination recombinant human follicle-stimulating hormone plus recombinant human luteinizing hormone (r-hFSH/r-hLH). The safety and tolerability of the two formulations were also assessed. Healthy premenopausal women were randomized to one of two crossover dosing schedules. Subjects in Treatment Sequence 1 received a single subcutaneous dose (900/450 IU r-hFSH/r-hLH) of the liquid formulation of r-hFSH/r-hLH on Day 1 of Dose Period 1 and, after a washout period of at least 14 days, a single subcutaneous dose (900/450 IU r-hFSH/r-hLH) of the freeze-dried formulation of r-hFSH/r-hLH (reconstituted in water for injection prior to administration) on Day 1 of Dose Period 2. Subjects in Treatment Sequence 2 received the treatments in reverse order. The primary endpoints were AUC_0–_*_**t**_* (area under the serum concentration–time curve from time 0 to the time of the last quantifiable concentration) and *C*_max_ (maximum serum concentration) for FSH and LH, both baseline corrected. A total of 34 subjects were randomized, and 22 subjects were included in the bioequivalence evaluation. Overall, the mean observed PK profiles and individual PK parameters were comparable for the liquid and freeze-dried formulations, although a median difference in the *t*_max_ (time to reach maximum observed concentration) of FSH of ~4.5 h was observed between the formulations. The calculated 90% confidence intervals of the mean liquid formulation/freeze-dried formulation ratios for *C*_max_ and AUC_0–_*_**t**_* were within the bioequivalence range (80–125%) for both LH and FSH, confirming bioequivalence between the two formulations. The safety and tolerability profiles of the two formulations were similar. The liquid formulation can, therefore, be expected to provide the same efficacy as the freeze-dried formulation, with no differences in tolerability.

## Introduction

Pergoveris^®^ (Merck KGaA, Darmstadt, Germany) is a fixed-dose, fixed-ratio (2:1) combination of recombinant human follicle-stimulating hormone (r-hFSH; 150 IU) plus recombinant human luteinizing hormone (r-hLH; 75 IU) indicated for the stimulation of follicular development in adult women with severe FSH and LH deficiency defined by an endogenous serum LH level <1.2 IU/L (1). r-hFSH/r-hLH is administered daily by subcutaneous injection with a recommended starting dose of 150 IU r-hFSH + 75 IU r-hLH and treatment individualized according to each patient’s response (1).

Recombinant human follicle-stimulating hormone/r-hLH is currently available as a freeze-dried powder provided in vials containing 150 IU r-hFSH + 75 IU r-hLH that are filled-by-mass, rather than filled-by-bioassay (1), as this enables greater consistency in activity between batches, which increases dosing precision (2, 3). However, the freeze-dried formulation has to be reconstituted with the provided solvent prior to subcutaneous injection using a syringe, and multiple vials might need to be reconstituted for a single prescribed dose (1). This reconstitution increases the number of steps required to prepare r-hFSH/r-hLH for injection and necessitates use of a syringe and vial.

A novel liquid formulation of r-hFSH/r-hLH was developed that does not require reconstitution and can be delivered subcutaneously using a prefilled, multi-dose pen injector, similar to the one currently available for GONAL-f^®^ (follitropin alfa; Merck KGaA, Darmstadt, Germany) and Ovidrel^®^ (rhCG; Merck KGaA, Darmstadt, Germany). Studies of this pen injector have demonstrated that health-care professionals find the pen injector easy to teach to use and that both health-care professionals and patients find the pen injector easy to learn to use, and can use it safely and effectively (4, 5). The pen injector provides reassurance that the correct dose has been administered, displaying feedback on the dose administered, and as there is no need for reconstitution, concern about correct preparation is reduced (4, 6). Furthermore, the pen injector has been demonstrated to reliably dispense accurate doses under a range of conditions, including cold, standard and warm atmospheres, and subsequent to freefall, vibration, dry-heat, cold-storage, and shipping preconditioning (7). These reasons, together with the discrete design of pen injectors and the potential to use a smaller gauge needle mean that pen injectors are a preferred option compared with needle and syringe, and liquid formulations are, therefore, desirable (8). These benefits have been observed following the development of liquid formulations of other fertility drugs (9, 10).

The European Medicines Agency (EMA) approved the liquid formulation of r-hLH/r-hFSH on 10 May 2017. In the current study, we assessed the bioequivalence, safety, and overall tolerability of the liquid and freeze-dried formulations of fixed-dose, fixed-ratio (2:1) r-hFSH/r-hLH.

## Materials and Methods

This was a Phase I, open-label, randomized, two-period, two-sequence crossover study (ClinicalTrials.gov NCT02317809; EudraCT number 2014-003506-32). The study was approved by the local ethics committee (Westminster Research Ethics Committee; Manchester, United Kingdom; 14/LO/2052) and conducted in accordance with the EMA guidelines on bioequivalence (11), the clinical study protocol, the International Council for Harmonization–Good Clinical Practice, and any additional applicable regulatory requirements. All subjects provided written informed consent prior to any study-related procedures.

### Study Design

**Figure 1:**
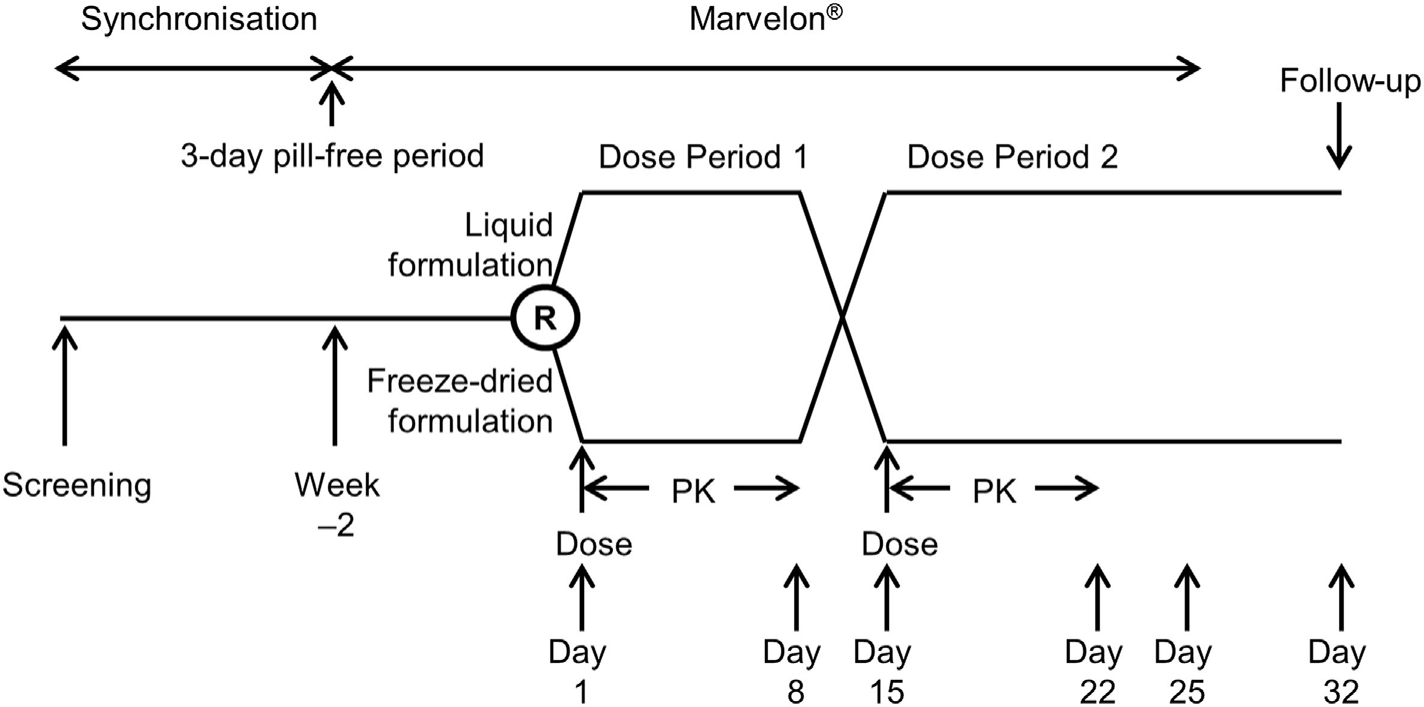
Study design. PK, pharmacokinetic; R, randomization.

The study design is shown in Figure 1. Following screening, subjects entered a synchronization period of 15–21 days to align the pill-free period, remaining on their own combined oral contraceptive pill (OCP) during this period. Subjects were synchronized in groups to align their pill-free period, with all subjects in each group discontinuing their own combined OCP on the same day. Following the synchronization period, subjects discontinued their combined OCP for 3 days, and on the morning after the 3-day pill-free period started taking Marvelon^®^ (150 µg desogestrel and 30 µg ethinylestradiol; Merck, Sharp & Dohme Ltd., Hoddesdon, UK) daily for 14–17 days. Marvelon had previously been used successfully to suppress endogenous FSH and LH levels in a bioequivalence study comparing freeze-dried r-hFSH/r-hLH filled-by-mass and filled-by-bioassay (12).

Subjects who met downregulation criteria (Appendix 1 in Supplementary Material) with Marvelon were randomized in a sequential order (using a randomization schedule) to one of two treatment sequences: subjects in Treatment Sequence 1 received a single dose of the liquid formulation of r-hFSH/r-hLH administered subcutaneously on Day 1 of Dose Period 1 and, after a washout period of at least 14 days, a single dose of the freeze-dried formulation of r-hFSH/r-hLH administered subcutaneously (after reconstitution) on Day 1 of Dose Period 2, while subjects in Treatment Sequence 2 received freeze-dried and liquid formulations in the opposite order.

Subjects remained on Marvelon until Day 11 of Dose Period 2 then returned to their own combined OCP. A follow-up visit was scheduled for 18 (±3) days after the last dose of r-hFSH/r-hLH.

### Subjects

Healthy premenopausal women aged 18–40 years (inclusive) taking a combined OCP for ≥1 year prior to screening were eligible for enrollment. Other inclusion and exclusion criteria are detailed in Appendix 1 in Supplementary Material.

### Study Treatments

The dose of r-hFSH/r-hLH used in this study was selected based on the results of a previous bioequivalence study that found that the lowest dose to ensure adequate serum levels to enable full characterization of the PK profile of r-hLH was 450 IU (12).

The liquid formulation of r-hFSH/r-hLH was presented in a prefilled 3 mL cartridge containing 900 IU r-hFSH and 450 IU r-hLH dissolved in 1.44 mL of water for injection. The cartridge was preassembled into a disposable pen injector intended for subcutaneous injection of multiple doses. The maximum dose that could be injected in a single injection with the pen injector was 450 IU r-hFSH and 225 IU r-hLH, and two injections were, therefore, required to give the full dose. The freeze-dried formulation was provided in vials containing 150 IU r-hFSH and 75 IU r-hLH. Three vials were consecutively reconstituted in 0.8 mL water to fill a single syringe for injection, and this was repeated so that two syringes were prepared for injection to provide 900 IU r-hFSH and 450 IU r-hLH.

A single dose of 900 IU r-hFSH and 450 IU r-hLH was administered subcutaneously on Day 1 of each dose period (Appendix 2 in Supplementary Material.), following an overnight fast, with doses injected into the abdomen.

### Endpoints

The primary PK endpoints were AUC_0–_*_t_* (area under the serum concentration–time curve from time 0 to the time of the last quantifiable concentration) and *C*_max_ (maximum serum concentration) for FSH and LH. In case of predose values, the PK parameters were baseline-adjusted. These parameters were then used for the determination of bioequivalence. Secondary PK endpoints for FSH and LH included AUC_0–∞_ (area under the serum concentration–time curve from time 0 extrapolated to infinity), *t*_max_ (time to reach maximum observed concentration), *t*_½_ (terminal half-life), CL/F (apparent serum clearance), and V_Z_/F (apparent volume of distribution during the terminal phase).

Safety was evaluated from when the subject was initially included in the study until the follow-up visit on Day 49 ± 3 days of the Marvelon cycle. Serious adverse events that might potentially be related to study treatment could be reported whenever they occurred; irrespective of the time elapsed since the last administration of study treatment. The following safety and tolerability endpoints were investigated: incidence and severity of treatment-emergent adverse events (TEAEs; any adverse events occurring after treatment with Marvelon or r-hFSH/r-hLH); vital signs and 12-lead electrocardiogram (ECG); routine hematology, clinical chemistry, and urinalysis; and local tolerability. Adverse events were categorized according to the Medical Dictionary for Regulatory Activities coding system, tabulated and listed by treatment and ethnic group, and analyzed by severity and relationship to drug. Laboratory and vital signs were descriptively summarized and shifts from baseline calculated. Local tolerability was evaluated by local reaction (including redness, swelling, bruising, and itching) evaluated by the investigator and the severity of pain evaluated using a visual analog scale (from “no pain” to “maximum pain”) by subjects. Any TEAE that occurred during a washout between treatment periods (i.e., time after end of the preceding treatment period but before start of treatment in the next treatment period) was attributed to the treatment given in the preceding period. Anti-drug antibody samples were taken before each dosing, 8 days after each dosing and at follow-up (18 ± 3 days after the last dose of r-hFSH/r-hLH; Figure 1).

PK endpoints were analyzed in the PK analysis set (detailed in Appendix 3 in Supplementary Material.) and sensitivity analyses were conducted in an extended PK analysis set (detailed in Appendix 3 in Supplementary Material.). A sensitivity analysis of subjects in the PK analysis set with baseline FSH and LH levels ≤0.05*C_max_ was also conducted. Safety was analyzed in the safety analysis set, which included all randomized subjects who received at least one dose of r-hFSH/r-hLH.

### Statistical Analyses

Assuming intra-subject coefficients of variation (CVs) of 14 and 11% for *C*_max_ and AUC_0–_*_t_*, respectively, for FSH, and 21 and 17%, respectively, for LH, 30 evaluable subjects were required to provide ≥90% power to demonstrate bioequivalence. These intra-subject CVs were selected based on data from a previous trial (Data on file). Allowing for a dropout rate of 20%, 38 subjects were proposed for randomization to treatment. Due to a low number of subjects who fulfilled the inclusion/exclusion criteria and a higher than expected number of subjects not downregulating sufficiently on Marvelon, the protocol was amended so that recruitment was stopped after 34 subjects had been randomized. A *post hoc* power calculation using the variability observed in the trial showed an 87% power to demonstrate bioequivalence with 22 subjects.

Serum PK variables were listed and summarized descriptively and all endpoints were baseline corrected, with baseline values below the lower limit of quantification set to 0. The log-transformed primary PK endpoints (AUC_0–_*_t_* and *C*_max_) for FSH and LH were analyzed using a linear mixed-effects model with treatment sequence, dose period, and treatment as fixed effects and subject as a random effect. Mean [90% confidence interval (CI)] treatment differences were estimated for all four primary endpoints and these were translated into ratios (liquid/freeze-dried formulation) following back-transformation. If the 90% CIs for all four ratios were within the range of 80–125%, then the two formulations were considered bioequivalent. For *t*_max_, the Hodges–Lehmann estimates for the difference between the liquid and freeze-dried formulations and the corresponding 90% CIs were computed. The incidence of TEAEs was summarized descriptively by treatment and System Organ Class/preferred term.

Five sensitivity analyses (secondary analyses) were conducted to explore the effect of baseline FSH and LH levels on the bioequivalence result and are listed in Appendix 4 in Supplementary Material.

Safety was evaluated as the frequency and percentage of subjects with any TEAEs, any TEAEs related to the trial drug, any serious TEAEs, any severe TEAEs, any TEAEs leading to study discontinuation, any TEAEs leading to study-drug discontinuation, and TEAEs with outcome of death (if any). The frequency and percentage was also evaluated.

## Results

### Subject Disposition

**Figure 2:**
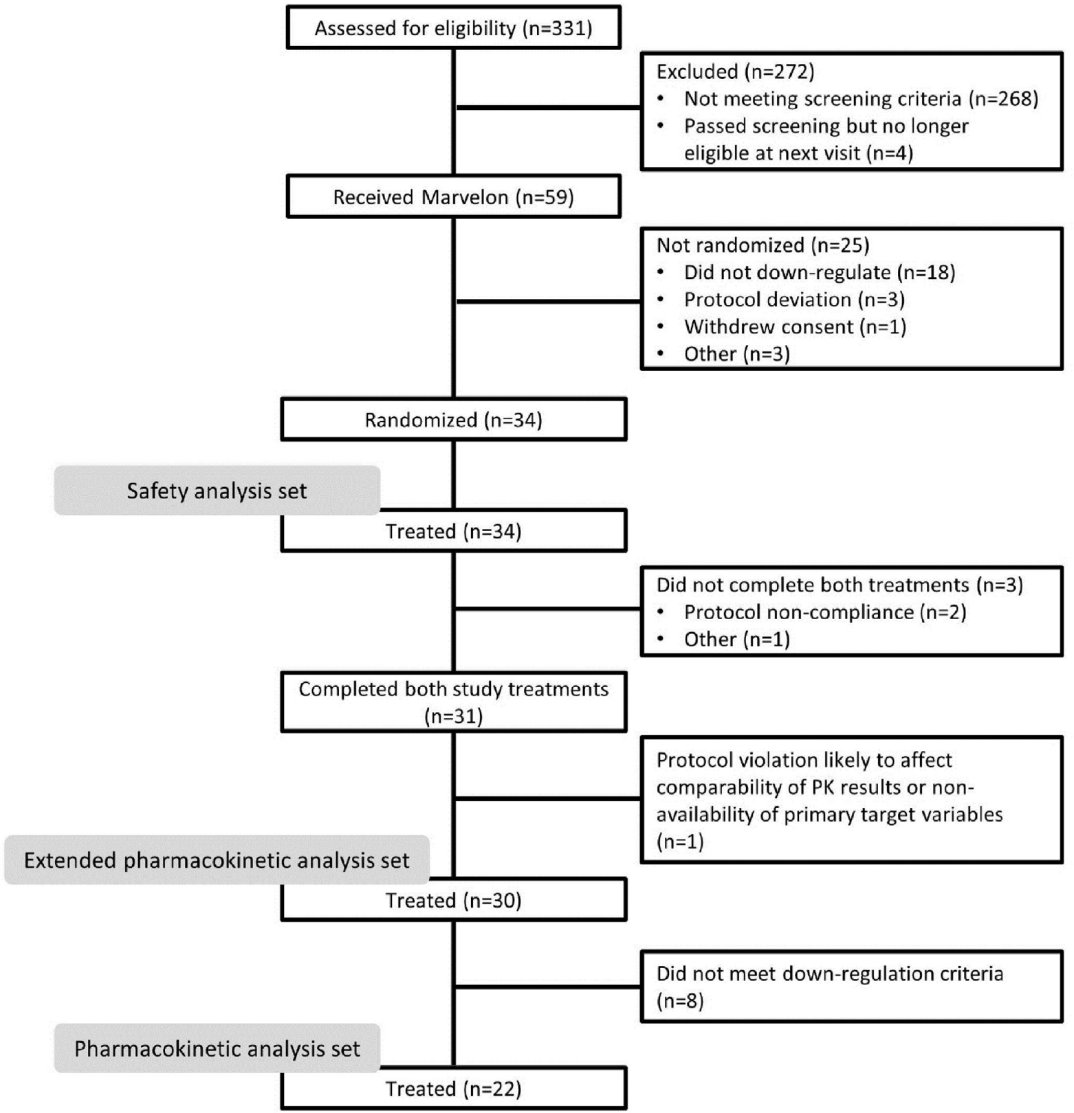
CONSORT flow diagram.

Overall, 331 subjects were screened (Figure 2), of whom 59 received Marvelon for pituitary suppression. Owing to difficulties recruiting subjects who met the inclusion criteria, in addition to a higher than expected number of subjects not downregulating sufficiently on Marvelon, 34 subjects (baseline characteristics shown in Table 1) were subsequently randomized. Of these, 31 subjects completed the study with three subjects discontinuing the study prematurely; treatment was terminated in two subjects owing to protocol noncompliance, and one subject was withdrawn prior to Dose Period 2 due to multiple follicles on transvaginal ultrasound with features of PCOS.

**Table 1:** Baseline characteristics.

	All subjects (*n* = 34)
Ethnicity, *n* (%)	
White	29 (85.3)
Black/African American	2 (5.9)
Asian	1 (2.9)
Mixed	2 (5.9)
Age (years), mean (SD)	28.6 (5.9)
Height (cm), mean (SD)	163.3 (6.8)
BMI (kg/m^2^), mean (SD)	22.6 (2.2)

*BMI, body mass index*.

The PK analysis set included 22 subjects; the main reason for exclusion from this set was failure to achieve baseline FSH and LH levels <1.0 IU/L, as measured in both screening and PK assays. The extended PK analysis set included 30 subjects, of whom three had baseline FSH and LH levels ≥10 IU/L. The safety analysis set included 34 subjects (liquid formulation, *n* = 34; freeze-dried formulation, *n* = 31).

### Pharmacokinetic Analysis

The results for the primary endpoints of the bioequivalence analysis are summarized in Table 2. The calculated 90% CIs of the mean liquid formulation/freeze-dried formulation ratios for *C*_max_ and AUC_0–_*_t_* were within the bioequivalence range of 80–125% for both LH and FSH, confirming the bioequivalence of the two formulations. The sensitivity analyses further supported bioequivalence (Table S1 in Supplementary Material); they also demonstrated that successful downregulation is an essential prerequisite for PK trials similar to the one described here.

**Table 2:** Bioequivalence results.

Parameter	Formulation	*N*	Geometric-least squares mean	Ratio^a^ (%)	90% CI of ratio	Intra-subject CV (%)
**Follicle-stimulating hormone**						
AUC_0–_*_t_*_,adj_ (IU·h/L)	Liquid	22	3,174.8	114.45	110.87–118.15	6.0
	Freeze dried	22	2,774.0			
*C*_max,adj_ (IU/L)	Liquid	22	47.94	112.67	106.44–119.27	10.8
	Freeze dried	22	42.55			
**Luteinizing hormone**						
AUC_0–_*_t_*_,adj_ (IU·h/L)	Liquid	22	210.8	106.99	101.42–112.86	10.1
	Freeze dried	22	197.1			
C_max,adj_ (IU/L)	Liquid	22	10.17	103.27	93.16–114.47	19.7
	Freeze dried	22	9.84			

*All data are baseline corrected*.

*^a^Liquid formulation/freeze-dried formulation*.

*adj, adjusted (baseline corrected); AUC_0–_*_t_*, area under the serum concentration–time curve from time 0 to the time of the last quantifiable concentration; CI, confidence interval; *C*_max_, maximum serum concentration; CV, coefficient of variation*.

**Figure 3:**
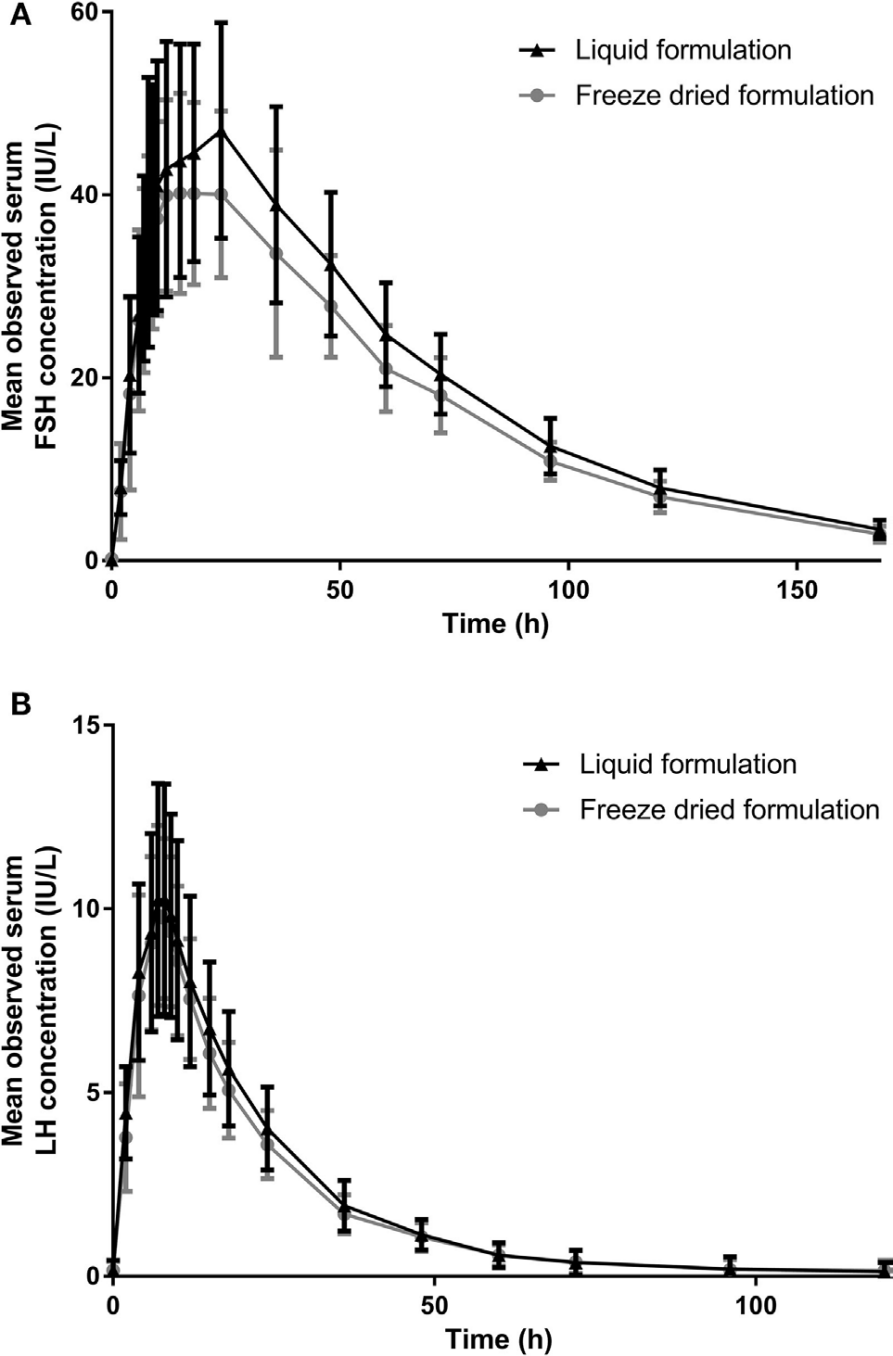
The mean (SD) observed serum **(A)** FSH and **(B)** LH concentration over time (PK analysis set). FSH, follicle-stimulating hormone; LH, luteinizing hormone; PK, pharmacokinetic.

Concentration–time profiles of FSH and LH in serum following the administration of the liquid and freeze-dried formulations of r-hFSH/r-hLH are shown in Figure 3 and the PK parameters are summarized in Table 3. Overall, the mean observed concentration–time profiles and PK parameters were comparable for the liquid and freeze-dried formulations. However, non-parametric statistical comparison revealed that the median difference in *t*_max_ between the liquid and freeze-dried formulations of FSH was about 4.5 h. This difference was statistically significant but did not result in statistically significant differences in exposure (AUC_0–_*_t_*_,adj_ and *C*_max,adj_) between the two formulations. A difference in *t*_max_ was also observed between the liquid and freeze-dried formulations for LH, this difference was about 0.5 h and not statistically significant.

**Table 3:** Pharmacokinetic parameters of follicle-stimulating hormone and luteinizing hormone after subcutaneous administration of either the liquid or freeze-dried formulation of fixed-ratio recombinant human follicle-stimulating hormone/recombinant human luteinizing hormone.

Parameter	Follicle-stimulating hormone		Luteinizing hormone	
	Liquid formulation (*N* = 22)	Freeze-dried formulation (*N* = 22)	Liquid formulation (*N* = 22)	Freeze-dried formulation (*N* = 22)
C_max,adj_ (IU/L)	47.92 (27.3) 27.5−88.3	42.55 (29.0) 22.6−77.6	10.126 (31.1) 6.00−18.58	9.782 (24.1) 6.38−17.78
AUC_0–_*_t_*_,adj_ (IU·h/L)	3,187.4 (24.3) 1,921−5,200	2,775.4 (22.0) 1,809−4,242	210.4 (25.8) 136−333	195.2 (22.5) 122−287
AUC_0–∞,adj_ (IU·h/L)	3,366.6 (24.2) 2,044−5,425	2,912.1 (22.2) 1,926−4,461	216.1 (25.2)^b^ 143−332	201.1 (21.2) 133−292
*t*_max_ (h)^a^	23.983 8.23−36.00	16.575 9.00−36.12	8.000 6.03−12.00	7.725 3.98−10.03
*t*_½_ (h)	36.87 (14.2) 30.2−55.3	35.31 (10.0) 30.8−45.6	12.507 (17.1)^b^ 9.27−18.92	13.608 (25.5) 8.13−24.57
CL/F (L/h)	0.2,673 (24.2) 0.166−0.440	0.3,091 (22.2) 0.202−0.467	2.082 (25.2)^b^ 1.36−3.16	2.238 (21.2) 1.54−3.38
V_Z_/F (L)	14.219 (27.1) 8.71−27.08	15.742 (21.4) 9.78−26.44	37.57 (28.8)^b^ 22.2−57.7	43.93 (32.3) 27.0−91.8

*All data are given as geometric mean [geometric coefficients of variation (%)] and range unless otherwise stated*.

*^a^Median and range*.

*^b^N = 21*.

*adj, adjusted (baseline corrected); AUC_0–_*_t_*, area under the serum concentration–time curve from time 0 to the time of the last quantifiable concentration; AUC_0–∞_, AUC from time 0 extrapolated to infinity *C*_max_, maximum serum concentration; *t*_max_, time to reach maximum observed concentration; *t*_1/2_, terminal half-life; CL/F, apparent serum clearance; V_Z_/F, apparently volume of distribution during the terminal phase*.

### Safety

Treatment with both the liquid and freeze-dried formulations of r-hFSH/r-hLH was well tolerated, with no new safety concerns identified. A total of 41 TEAEs were reported during the study [liquid formulation:12/34 (35.3%) subjects reported 17 TEAEs; freeze-dried formulation: 14/31 (45.2%) subjects reported 24 TEAEs; Table 4]. The safety and tolerability profile of the liquid formulation was similar to that of the freeze-dried formulation. No deaths were reported during the study and no subjects withdrew because of TEAEs. The most commonly reported TEAE was headache; reported by six (17.6%) and four (12.9%) subjects receiving the liquid and freeze-dried formulation, respectively.

**Table 4:** Comparison of treatment-emergent adverse events after receiving the liquid or freeze-dried formulations of recombinant human follicle-stimulating hormone/recombinant human luteinizing hormone.

	Recombinant human follicle-stimulating hormone/recombinant human luteinizing hormone			
	Liquid formulation (*N* = 34)		Freeze-dried formulation (*N* = 31)	
	Subjects, *N* (%)	Events, *N*	Subjects, *N* (%)	Events, *N*
TEAEs (related and not related)	12 (35.3)	17	14 (45.2)	24
Treatment-related TEAEs	5 (14.7)	7	4 (12.9)	6
Serious AEs	1 (2.9)	1^a^	0 (0.0)	0
Severe TEAEs	1 (2.9)	1^a^^,^^b^	0 (0.0)	0
TEAEs leading to study discontinuation	0 (0.0)	0	0 (0.0)	0
**TEAEs occurring in >10% of subjects in either group**				
Headache				
All cases	6 (17.6)	6	4 (12.9)	4
Assessed as related	4 (11.8)	4	2 (6.5)	2
Catheter site related reaction	1 (2.9)	1^a^	4 (12.9)	5^a^
**Local tolerability**				
Mild redness				
5 min after injection	4 (11.8)	4	5 (16.1)	5
1 h after injection	2 (5.9)	2	1 (3.2)	1
2 h after injection	0	0	1 (3.2)	1
4 h after injection	0	0	1 (3.2)	1
6 h after injection	0	0	1 (3.2)	1
Mild itching				
5 min after injection	0	0	1 (3.2)	1
1 h after injection	0	0	0	0
2 h after injection	0	0	0	0
4 h after injection	0	0	0	0
6 h after injection	1 (2.9)	1	0	0
Swelling	0	0	0	0
Bruising	0	0	0	0

*AE, adverse event; TEAE, treatment-emergent adverse event*.

*^a^Not related to the study drug*.

*^b^This is the same event as the serious AE*.

One serious AE, classified as a severe TEAE, was reported for a subject who informed the investigators during a follow-up visit (Day 58) that she had been diagnosed with non-Hodgkin’s lymphoma. The subject received the freeze-dried formulation followed by the liquid formulation and had completed all study-related assessments at the time of the diagnosis, but on consideration of the medical history and subsequent evaluations, this serious AE was considered to be unrelated to treatment with either r-hFSH/r-hLH or Marvelon.

Injections with both formulations of r-hFSH/r-hLH were well tolerated and injection site assessments did not reveal any notable differences between them (Table 4). All of the catheter site-related reactions were not related to r-hFSH/r-hLH administration, rather they were related to the catheter (venule) inserted for blood sampling.

None of the laboratory, vital sign or 12-lead ECG assessments showed any relevant changes after treatment and none of the individual values were clinically significant.

All subjects were negative for anti-LH anti-drug antibodies and only two subjects showed positive signs for anti-FSH anti-drug antibodies. These signs were present at baseline, and only low titers (1.0 and 1.7, respectively) were reported. Since only a minor and transient increase was observed in a single subject at approximately 14 days after the first dose, with no increase after subsequent doses, it was concluded that no relevant induction of anti-drug antibodies had occurred.

## Discussion

This randomized, two-way crossover trial in healthy female subjects demonstrated, in the primary PK analysis set, the bioequivalence of the liquid and freeze-dried formulations of r-hFSH/r-hLH when given as a single-dose split across two injections. Furthermore, no new safety concerns were identified.

A difference in the *t*_max_ of FSH was observed between the liquid and freeze-dried formulations. This difference was estimated by non-parametric statistical comparison to be a median of about 4.5 h, but did not result in significant differences in exposure (AUC_0–_*_t_*_,adj_ and *C*_max,adj_) between the two formulations. Furthermore, as shown in Figure 1, the overall mean concentrations of FSH were generally slightly higher after administration of the liquid formulation than after administration of the freeze-dried formulation. This is considered a minor variation within the limits of bioequivalence.

As summarized in Table 3, the AUC_0–_*_t_* and *C*_max_ of FSH were 14 and 13% higher, respectively, after administration of the liquid formulation. The higher concentrations of FSH and LH after administration of the liquid formulation may be related to the difficulty of extracting all of the freeze-dried material from the vials. It is unlikely that these increases are of clinical relevance as, in general, the choice of initial dose and subsequent dose adjustments are not made on the basis of serum FSH levels, but rather on clinical response. Therefore, minor differences in FSH levels of the magnitude observed are unlikely to have a relevant impact on outcomes. Furthermore, high inter- and intra-patient variability has been observed in response to identical doses of FSH, and results in no clear differences in clinical response (13), and no correlation could be described between pharmacodynamic effects, serum FSH or LH levels (14).

As reconstitution is not required with the liquid formulation, this should reduce both the burden for the patient and the risk of administration errors (15). Furthermore, as the liquid formulation can be delivered using a prefilled, multi-dose pen, which is ready to use once a needle has been attached, the number of preparatory steps required before injection are further reduced, increasing convenience and reducing potential for error. Pen injectors have also been demonstrated to be more accurate, easier-to use, more discreet and less stressful to use compared with syringe and drug vial (6, 15–22). This should, therefore, increase convenience of use and may improve adherence (i.e., taking the therapy according to the agreed treatment plan) (21).

The main limitation of the study was that randomization was stopped before 38 subjects entered the study, owing to difficulties recruiting eligible subjects fulfilling the inclusion/exclusion criteria and a higher than expected number of subjects not down-regulating sufficiently on Marvelon. Of the 34 randomized and treated subjects, four did not have appropriate PK parameters in both periods and had to be excluded, and eight appeared to be inappropriately included in the study since their LH levels did not fulfill the required down-regulation criterion of being below 1.0 IU/L. However, due to the observed low CV% in the study, 22 subjects were appropriate to conclude bioequivalence of the two formulations, providing 87% power for the determination of bioequivalence. Bioequivalence was further supported in four out of the five sensitivity analyses conducted.

Evidence from previous PK studies of r-hFSH or r-hLH in healthy females, as well as in women with infertility, demonstrated that both r-hFSH and r-hLH exhibit linear pharmacokinetics over a broad range of doses administered either intravenously or subcutaneously (1). Furthermore, it is well established that the single-dose PK profiles of r-hFSH and r-hLH do not differ when administered either alone or as a fixed-dose combination. The linear PK of both analytes, as well as the absence of an interaction between LH and FSH, allows the translation of the PK findings of the high dose fixed combination of 900/450 IU r-hFSH/r-hLH from this study to the therapeutic dose, i.e., 150 IU r-hFSH plus 75 IU r-hLH.

In conclusion, this study demonstrated the bioequivalence of the liquid and freeze-dried formulations of fixed-ratio (2:1) r-hFSH/r-hLH. Furthermore, single-dose administration of both formulations was well tolerated, with no new safety signals identified. The liquid formulation can, therefore, be expected to provide the same efficacy as the freeze-dried formulation, with no differences in tolerability.

## Ethics Statement

This study was carried out in accordance with the recommendations of with European Medicines Agency guidelines on bioequivalence (11) with written informed consent from all subjects. All subjects gave written informed consent in accordance with the Declaration of Helsinki. The protocol was approved by the local ethics committee (Westminster Research Ethics Committee, Manchester, United Kingdom; 14/LO/2052).

## Author Contributions

WB, OY, and PW were involved in the study conception and design, analysis, and interpretation of data and critical review and revision of the manuscript. All authors provided final approval to submit the manuscript.

## Conflict of Interest Statement

WB is an employee of the Merck Institute for Pharmacometrics, Lausanne, Switzerland, an affiliate of Merck KGaA, Darmstadt, Germany. OY and PW are employees of Merck KGaA, Darmstadt, Germany.
