# Supplementary material for: Open-Label, Randomized, Two-Way, Crossover Study Assessing the Bioequivalence of the Liquid Formulation versus the Freeze-Dried Formulation of Recombinant Human FSH and Recombinant Human LH in a Fixed 2:1 Combination (Pergoveris®) in Pituitary-Suppressed Healthy Women
Source: Front Endocrinol (Lausanne). 2018 Jan 11;8:371. doi: 10.3389/fendo.2017.00371 (PMC5768635; doi:10.3389/fendo.2017.00371)
Supplement: Supplementary file 1 [file data_sheet_1.PDF]

Supplementary Material

Open-label, randomized, two-way, crossover study assessing the bioequivalence of the liquid formulation versus the freeze-dried formulation of recombinant human FSH and recombinant human LH in a fixed 2:1 combination (Pergoveris®) in pituitary-suppressed healthy women

Wilhelmina Bagchus*, Özkan Yalkinoglu, Peter Wolna

First Author*, Co-Author, Co-Author

*** Correspondence:** Wilhelmina Bagchus wilhelmina.bagchus@merckgroup.com

1 Supplementary Appendices

1.1 Appendix 1 – Inclusion and exclusion criteria

Down-regulation criteria were luteinizing hormone (LH) <1.0 IU/L, follicle-stimulating hormone (FSH) <1.0 IU/L, estradiol <100 pg/mL [<366 pmol/L], absence of mature follicles and ≤ 12 immature follicles per ovary.

Inclusion criteria included healthy premenopausal women aged 18–40 years (inclusive) taking a combined OCP for ≥1 year prior to screening (and willing to recommence taking their own combined OCP from Day 11 of Dose Period 2) until follow-up with normal levels of FSH (<12 IU/L) and estradiol (<100 pg/mL); body weight ≥48 kg and body mass index (BMI) of 18.5–29.9 kg/m^2 (inclusive); non-smoking or having refrained from smoking for ≥6 months prior to screening and with a history of <10 pack-years; and negative pregnancy test at screening, before commencing on Marvelon and at admission to Dose Periods 1 and 2; normal cervical cytology assessment (Papanicolaou test score ≤II) within 12 months of screening.

Exclusion criteria included contraindications to combined OCPs or treatment with gonadotropins; history of tumours of the pituitary gland or hypothalamus; clinically significant abnormalities of the genital organs; unsuccessful down-regulation (i.e. LH ≥1.0 IU/L, FSH ≥1.0 IU/L, estradiol ≥100 pg/mL [≥366 pmol/L], absence of mature follicles or ≤12 immature follicles per ovary) with Marvelon prior to randomization; polycystic ovary syndrome [PCOS] defined by at least two of the following three criteria: >12 follicles with a mean diameter <10 mm in either ovary, ≥1 ovary of >10 mL volume, or increased androgen level (testosterone >0.6 μg/L); pregnant or breastfeeding; prior treatment with FSH- and/or LH-containing products; definite or suspected personal or family history of an adverse drug reaction or hypersensitivity to drugs with a similar chemical structure to FSH or LH; history or presence of asthma (with the exception of childhood asthma) or any serious allergy requiring hospitalization or prolonged systemic treatment; and use of drugs that might reduce the

effectiveness of combined OCPs from the start of Marvelon until the last pharmacokinetic (PK) sample.

1.2 Appendix 2 – Study drug dosing

The liquid formulation of r-hFSH/r-hLH was presented in a prefilled 3 mL cartridge containing 900 IU r-hFSH and 450 IU r-hLH dissolved in 1.44 mL of water for injection. The cartridge was preassembled into a disposable pen injector intended for subcutaneous injection of multiple doses. The maximum dose that could be injected in a single injection with the pen injector was 450 IU r-hFSH and 225 IU r-hLH, and two injections were therefore required to give the full dose. The freeze-dried formulation was provided in vials containing 150 IU r-hFSH and 75 IU r-hLH. Three vials were consecutively reconstituted in 0.8 mL water to fill a single syringe for injection, and this was repeated so that two syringes were prepared for injection to provide 900 IU r-hFSH and 450 IU r-hLH.

1.3 Appendix 3 – PK analysis sets

The PK analysis set included all randomized subjects who received both the liquid and freeze-dried formulations of r-hFSH/r-hLH and who fulfilled the following criteria: absence of relevant protocol violations with respect to factors likely to affect the comparability of the PK results; availability of C_{max} and $AUC_{0–t}$ data for FSH and LH for both formulations/dose periods; and successful FSH and LH down-regulation.

The extended PK analysis set included all randomized subjects who received both the liquid and freeze-dried formulations of r-hFSH/r-hLH and who fulfilled the following criteria: absence of relevant protocol violations with respect to factors likely to affect the comparability of the PK results, however baseline FSH or LH exclusion criteria were not applied; availability of C_{max} and $AUC_{0–t}$ data for FSH and LH for both formulations/dose periods.

1.4 Appendix 4 – Sensitivity analyses

1. PK analysis set (without baseline correction; n=22)
2. PK analysis set (with baseline as covariate, without baseline correction, n=22)
3. Extended analysis set (omitting condition at baseline, i.e. no baseline FSH or LH exclusion criteria applied; n=30)
4. Extended analysis set (relaxed condition at baseline, FSH and LH ≤10 IU/L; n=27)
5. Subjects with baseline ≤ 0.05*Cmax (n=17)

2 Supplementary Tables

Supplementary Table 1. Sensitivity analyses of bioequivalence results: **A)** unadjusted PK parameters (i.e. without baseline correction) in the PK analysis set (n=22); **B)** unadjusted PK parameters (i.e. without baseline correction) with baseline as covariate in the PK analysis set (n=22); **C)** adjusted PK parameters in the extended analysis set (n=30); **D)** adjusted PK parameters in subjects in the extended analysis set without high baseline FSH and LH values (both ≤10 IU/L; n=27); **E)** unadjusted PK parameters (i.e. without baseline correction) in subjects with baseline ≤ 0.05*C_{max} (n=17).

A)

Parameter	Formulation	N	Geometric-least squares mean	Ratio* (%)	90% CI of ratio	Intra-subject CV (%)
Follicle-stimulating hormone						
AUC_{0-t} (IU·h/L)	Liquid	22	3192.2	113.80	110.15, 117.57	6.2
	Freeze dried	22	2805.2			
C_{max} (IU/L)	Liquid	22	48.03	112.36	106.18, 118.90	10.7
	Freeze dried	22	42.75			
Luteinizing hormone						
AUC_{0-t}(IU·h/L)	Liquid	22	229.9	108.04	103.16, 113.16	8.8
	Freeze dried	22	212.8			
C_{max} (IU/L)	Liquid	22	10.34	103.44	93.41, 114.54	19.5
	Freeze dried	22	9.99			

*Liquid formulation/freeze-dried formulation

AUC_{0-t}, area under the serum concentration–time curve from time 0 to the time of the last quantifiable concentration; CI, confidence interval; C_{max}, maximum serum concentration; CV, coefficient of variation.

B)

Parameter	Formulation	N	Geometric-least squares mean	Ratio* (%)	90% CI of ratio	Intra-subject CV (%)
Follicle-stimulating hormone						
AUC_{0-t} (IU·h/L)	Liquid	22	3199.8	114.42	110.55, 118.42	6.2
	Freeze dried	22	2796.6			
C_{max} (IU/L)	Liquid	22	48.18	113.16	106.63, 120.09	10.7
	Freeze dried	22	42.58			
Luteinizing hormone						
AUC_{0-t}(IU·h/L)	Liquid	22	228.3	107.41	102.31, 112.77	9.1
	Freeze dried	22	212.5			
C_{max} (IU/L)	Liquid	22	10.29	103.06	93.06, 114.14	19.5
	Freeze dried	22	9.99			

*Liquid formulation/freeze-dried formulation

AUC_{0-t}, area under the serum concentration–time curve from time 0 to the time of the last quantifiable concentration; CI, confidence interval; C_{max}, maximum serum concentration; CV, coefficient of variation.

C)

**Parameter**	**Formulation**	**N**	**Geometric-least squares mean**	**Ratio* (%)**	**90% CI of ratio**	**Intra-subject CV (%)**
Follicle-stimulating hormone						
$AUC_{0–t, adj}$ (IU·h/L)	Liquid	30	3169.1	115.65	112.80, 118.57	5.7
	Freeze dried	30	2740.4			
$C_{max, adj}$ (IU/L)	Liquid	30	48.11	112.35	107.56, 117.36	9.9
	Freeze dried	30	42.82			
Luteinizing hormone						
$AUC_{0–t, adj}$ (IU·h/L)	Liquid	30	211.1	112.26	98.43, 128.03	30.5
	Freeze dried	30	188.0			
$C_{max, adj}$ (IU/L)	Liquid	30	10.59	103.43	92.65, 115.47	25.4
	Freeze dried	30	10.24			

All data are baseline corrected. *Liquid formulation/freeze-dried formulation

adj, adjusted (baseline corrected); $AUC_{0–t,}$ area under the serum concentration–time curve from time 0 to the time of the last quantifiable concentration; CI, confidence interval; C_{max}, maximum serum concentration; CV, coefficient of variation.

D)

Parameter	Formulation	N	Geometric-least squares mean	Ratio* (%)	90% CI of ratio	Intra-subject CV (%)
Follicle-stimulating hormone						
$AUC_{0–t, adj}$ (IU·h/L)	Liquid	27	3206.8	115.96	112.80, 119.22	5.9
	Freeze dried	27	2765.4			
$C_{max, adj}$ (IU/L)	Liquid	27	48.69	112.12	106.80, 117.71	10.4
	Freeze dried	27	43.43			
Luteinizing hormone						
$AUC_{0–t, adj}$ (IU·h/L)	Liquid	27	217.8	106.20	100.92, 111.75	10.9
	Freeze dried	27	205.1			
$C_{max, adj}$ (IU/L)	Liquid	27	10.69	105.85	96.94, 115.57	19.0
	Freeze dried	27	10.10			

All data are baseline corrected. *Liquid formulation/freeze-dried formulation

adj, adjusted (baseline corrected); $AUC_{0–t}$, area under the serum concentration–time curve from time 0 to the time of the last quantifiable concentration; CI, confidence interval; C_{max}, maximum serum concentration; CV, coefficient of variation.

E)

Parameter	Formulation	N	Geometric-least squares mean	Ratio* (%)	90% CI of ratio	Intra-subject CV (%)
Follicle-stimulating hormone						
$AUC_{0–t}$ (IU·h/L)	Liquid	17	3289.2	113.39	108.77, 118.20	6.6
	Freeze dried	17	2900.9			
C_{max} (IU/L)	Liquid	17	49.20	109.89	102.80, 117.47	10.6
	Freeze dried	17	44.77			
Luteinizing hormone						
$AUC_{0–t}$(IU·h/L)	Liquid	17	214.4	108.68	102.29, 115.47	9.7
	Freeze dried	17	197.3			
C_{max} (IU/L)	Liquid	17	10.00	102.57	90.84, 115.81	19.5
	Freeze dried	17	9.75			

*Liquid formulation/freeze-dried formulation

$AUC_{0–t}$, area under the serum concentration–time curve from time 0 to the time of the last quantifiable concentration; CI, confidence interval; C_{max}, maximum serum concentration; CV, coefficient of variation.
